# Effects of dietary supplementation of probiotic *Enterococcus faecium* on growth performance and gut microbiota in weaned piglets

**DOI:** 10.1186/s13568-019-0755-z

**Published:** 2019-03-01

**Authors:** Chengjun Hu, Weigang Xing, Xiaohua Liu, Xiuzhu Zhang, Ke Li, Jia Liu, Baichuan Deng, Jinping Deng, Yong Li, Chengquan Tan

**Affiliations:** 1 Guangdong Provincial Key Laboratory of Animal Nutrition Control, Institute of Subtropical Animal Nutrition and Feed, College of Animal Science, South China Agricultural University, Guangzhou, 510640 China; 2 Newhope Liuhe Corp. Ltd, Beijing, 100102 People’s Republic of China

**Keywords:** Antibiotics, *Enterococcus faecium*, Growth performance, Microbiota, Weaned piglets, 16SrRNA gene

## Abstract

The adverse effects of antibiotics have attracted widespread attention, thus reducing the use of antibiotics in animal feed has become a very important issue in improving of the health of livestock. The effects of *Enterococcus faecium* (*E. faecium*) on growth performance and gut microbiota in weaned piglets were investigated in the present study. Piglets were randomly assigned to four treatments: a control group fed with a diet containing 75 mg/kg aureomycin (Diet 1 group) and three experimental groups fed with diets of 50 mg/kg aureomycin (Diet 2 group), 50 mg/kg aureomycin + 9 × 10^5^ CFU/g *E. faecium* (Diet 3 group), or 50 mg/kg aureomycin + 1.2 × 10^6^ CFU/g *E. faecium* (Diet 4 group). Their gut microbial communities were analyzed by sequencing the V3–V4 region of the 16SrRNA gene. The results showed that the final body weights and the average daily gain of the weaned piglets in the Diet 2 group were higher (*P* = 0.05) than those in the Diet 1 or Diet 3 group. Decreasing trends (*P *= 0.08) was observed in mortality rate in the Diet 3 and 4 group when compared with that in the Diet 1 group. Increases in the Sobs, Chao1, ACE, and Shannon indexes and a decrease in the Simpson index were observed at intervals from day 1 to 14 (*P* < 0.05). The Sobs, Chao1, and ACE indexes in the Diet 3 group were the lowest on day 14 (*P* < 0.05). The abundance of *Bacteroidetes* was increased and that of *Proteobacteria* was decreased from day 1 to 7, but both of them kept stable from day 7 to 14. Besides, the lowest abundance of *Fusobacteria*, *Lentisphaerae*, and *Planctomycetes* was observed on day 1 and the lowest abundance of *Actinobacteria* was observed on day 14 in the Diet 3 group (*P *< 0.05). Overall, these results suggest that the antibiotics and *E. faecium* interventions result in different changes in the gut microbiota, and a reduced antibiotics diet supplemented with 1.2 × 10^6^ CFU/g *E. faecium* does not affect the growth performance in weaned piglets.

**Electronic supplementary material:**

The online version of this article (10.1186/s13568-019-0755-z) contains supplementary material, which is available to authorized users.

## Introduction

Antibiotics have been widely used in the prevention of diarrhea and the improvement of the growth of livestock. However, the adverse effects of antibiotics, such as residues in meat products and the emergence of antibiotic-resistant bacteria (van den Bogaard and Stobberingh 2000), have attracted widespread attention. Thus, antibiotics are forbidden to be used as additives in animal feed in some countries (Casewell et al. 2003). In recent years, the use of antibiotics in animal feed has been gradually reduced in China, but is still sometimes available in animal feed. Therefore, it’s of great significance to find alternatives to antibiotics in animal feed to promote the development of livestock industry.

Probiotic feed additives have been proposed as alternatives to antibiotics due to their positive effects on hosts (Liu et al. 2014; Abhisingha et al. 2017; Yu et al. 2017). *Enterococcus faecium* is widely used as a probiotic supplement in feed. Previous studies showed a beneficial effect of probiotic *E. faecium* on diarrhea, growth performance, and microbiota composition (Zeyner and Boldt 2006; Bednorz et al. 2013; Wang et al. 2016; Lan and Kim 2017), suggesting that antibiotics may be replaced by *E. faecium*. However, some studies indicate that *E. faecium* treatment has no effects on body weight gain (Busing and Zeyner 2015), feed intake, or feed efficiency in piglets (Taras et al. 2006). Thus, the effects of *E. faecium* on growth performance in piglets remain highly controversial. Thus, further studies are needed to elucidate the mechanisms in the effect of *E. faecium*.

In recent years, the roles of gut microbiota have been extensively investigated and revealed (Kahrstrom et al. 2016; Sonnenburg and Backhed 2016). Symptoms of metabolic syndrome of the hosts such as obesity are closely associated with dysbiosis of the gut microbiota (Sen et al. 2017). The gut microbiota have a major impact on the health of piglets; for instance, the production of amino acids, the fermentation of carbohydrates, the maintenance integrity of the intestinal villi, and the protection from pathogenic bacteria (Gresse et al. 2017). The decrease in the population of *Lactobacillus* genus and the increase in the population of *Enterococcus* and *Escherichia coli* were observed in early weaning piglets (Wei et al. 2017). Moreover, changes in the microbial community structure are seen in piglets with intestinal disorders, such as diarrhea (Li et al. 2014). Obviously, gut microbiota are an important factor that affects the growth of piglets. The composition of the microbiota in the gastrointestinal tract varies between piglets fed with an antibiotics-supplemented diet and those fed with an antibiotics-free diet (Mu et al. 2017), which indicates that antibiotics-induced changes in the gut microbiota may lead to the changes in the growth of piglets (Andreas et al. 2016).

Although some studies have focused on the roles of antibiotics and *E. faecium* in the growth of piglets (Wang et al. 2013, 2016; Lan and Kim 2017), there’s still little information about the effects of a diet with reduced antibiotics and *E. faecium* supplementation on the growth and fecal bacterial community structure of animals. Early weaned piglets are exposed to several stress factors which make gut microbiota dramatically change and make the diarrhea increase without antibiotics treatment (Vondruskova et al. 2010; Li et al. 2017). Therefore, this study is conducted to evaluate the effects of antibiotics and *E. faecium* on growth performance and gut microbiota in weaned piglets.

## Materials and methods

### Animals and experimental treatments

The experimental design and procedure presented in this study are reviewed and approved by the Animal Care and Use Committee of the South China Agricultural University.

364 weaned piglets (Duroc × Landrace × Large White) with an initial body weight of 7.03 ± 0.03 kg were randomly assigned to four treatments with seven pens, and each pen contains 13 weaned piglets. The piglets are fed with water and a corn and soybean meal-based diet (Table 1) ad libitum through a nipple drinker and a feeder. The piglets in the control group were fed with a basal diet containing 75 mg/kg aureomycin (Diet 1 group), and those in the three experimental groups were fed a basal diet with the following supplements: 50 mg/kg aureomycin (Diet 2 group), 50 mg/kg aureomycin + 9 × 10^5^ CFU/g *E. faecium* (Diet 3 group), or 50 mg/kg aureomycin + 1.2 × 10^6^ CFU/g *E. faecium* (Diet 4 group). *E. faecium* (China Center for Type Culture Collection, Wuhan, China, CCTCC No. M2011031, 3 × 10^9^ CFU/g) was provided by Huada-real Technology Co., Ltd. (Wuhan, China). The experiment was performed for 14 days.

**Table 1:** Composition and nutrient levels of the basal diet (g/kg, as-fed basis)

Ingredients	Content
Corn	576.7
Soybean oil	20
Extruded full-fat soybean	60
Soybean meal	172.5
Spray-dried plasma protein	60
Whey powder dried	80
Salt	1.4
CaHPO_4_	16
Lys	3.9
Met	2.5
Thr	2
Premix^a^	5
Chemical composition^b^	
Digestible energy, kcal/kg	3508
CP^c^, %	20.3
CF^d^, %	2.3
Crude ash, %	4.5
Ca, %	0.7
Total P, %	0.7
Salt, %	0.5
Total Lys, %	1.4

^a^Premix provided for 1 kg of complete diet: vitamin A, 11,750 IU; vitamin D_3_, 1500 IU; vitamin E, 50 IU; vitamin K, 1.75 mg; vitamin B_1_ 1 mg; vitamin B_2_, 10 mg; vitamin B_6_, 1 mg; vitamin B_12_ 27.5 mg; niacin, 38 mg; calcium pantothenate, 35.75 mg; choline chloride 750 mg; biotin 100 μg; folic acid 0.5 mg; Cu as copper sulfate, 125 mg; I as kalium jodatum, 0.75 mg; Fe as iron sulfate, 152.5 mg; Mn as manganese oxide, 35 mg; Mg as magnesium sulfate, 125 mg; Zn as zinc sulfate, 137.5 mg

^b^Calculated values

^c^Crude protein

^d^Crude fiber

### Sample collection and measurements

Initial body weight and final body weight of the piglets were measured at the age of 21 days (experimental day 1) and 35 days (experimental day 14) to calculate the average daily weight gain. The amounts of feed offered and refused were recorded every day to confirm the individual daily feed intake, and the feed efficiency was calculated by the weight gain/feed intake ratio based on the data of feed intake and body weight. The diarrhea rate was calculated according to the following formula (Hu et al. 2017): *A*/(*B* × *C*), where *A* is the number of piglets with diarrhea in the pen, *B* is the total number of piglets in the pen, and *C* is the number of experimental days.

### DNA extraction and 16SrRNA gene sequencing

72 fecal samples were collected after feeding, with 18 samples collected per group, and 6 samples collected per period (on day 1, 7, and 14, respectively). Total genomic DNA were extracted from fecal samples using QIAamp DNA Stool Mini Kit (Qiagen, Hilden, Germany) following the instructions. A NanoDrop ND-1000 system (Thermo Fisher, Wilmington, DE, USA) was used to measure the concentration of DNA. The V4 region of the 16SrRNA gene was amplified using primers 515F (5′-GTGCCAGCMGCCGCGGTAA-3′) and 806R (5′-GGACTACHVGGGTWTCTAAT-3′) (Zeng et al. 2017). Total reaction volume of 20 μL comprised 2 μL 2.5 mM dNTPs, 4 μL 5×FastPfu buffer (TransGen Biotech, Beijing, China), 0.4 μL FastPfu Polymerase, 0.8 μL of each primer, 1 μL DNA template, and 11 μL ddH_2_O. The PCR program included a 3-min incubation at 95 °C, followed by 27 cycles of denaturation at 95 °C for 30 s, and annealing and extension at 55 °C for 30 s and at 72 °C for 45 s. All samples examined in this study provided complete DNA samples, as agarose gels clearly showed the amplified products. After PCR amplification, amplicons were extracted from 1.2 agarose gels and purified using SanPrep DNA Gel Extraction Kit (Sangon Biotech, China). Purified amplicons were operated using paired-end sequencing by Illumina MiSeq. The instructions of the platform and the manufacturer were from a commercial service provider (BGI, Shenzhen, China). Sequences with an average phred score lower than 30, ambiguous bases, homopolymer runs exceeding 6 bp, primer mismatches, or sequence lengths shorter than 100 bp were removed. All the procedures except DNA extraction were conducted by the BGI Company.

### Bioinformatics analysis

The bioinformatics analysis will be carried out based on the sequencing data. The raw data were analyzed by QIIME (http://qiime.org/) (Caporaso et al. 2010) and FLASH (v1.2.11) (Magoc and Salzberg 2011), and were filtered to eliminate adapters and low-quality reads to obtain clean reads, and then overlapped paired-end reads were merged to create tags. The tags were clustered into operational taxonomic units (OTUs) with sequence similarity of 97% using USEARCH (v7.0.1090) (Edgar 2013). Representative OTU sequences were taxonomically classified by Ribosomal Database Project (RDP) Classifier trained on Greengene (V201305) reference database (DeSantis et al. 2006). Finally, alpha diversity was analyzed based on OTUs. Principal component analysis (PCA) plots of the dissimilarity metrics were also visualized using the R (v3.0.3). All the raw sequences were submitted to the NCBI Sequence Read Archive with an Accession Number of SAMN10234820-SAMN10234874.

### Statistical analysis

The growth performance, observed OTUs, and alpha diversity were statistically analyzed by repeated-measure one-way ANOVA using SPSS 17.0 (SPPS Inc., Chicago, IL, USA). Duncan’s multiple-range test and multivariate analysis of variance performed in the case of Mauchly’s test of Sphericity showed *P* > 0.05 and *P* < 0.05, respectively. The relative abundance at phylum and genus levels was statistically analyzed through non-parametric Kruskal–Wallis tests. The relationships between mortality diarrhea rate and diet were statistically analyzed through Chi squared test. Variations between different methods were considered statistically remarkable at *P* ≤ 0.05, with the trends toward significance indicated by 0.05 < *P* < 0.10.

## Results

### Growth performance

As shown in Table 2, compared with the Diet 1 and Diet 3 groups, the final body weight in the Diet 2 group increased (*P* = 0.05) by 4.13% and 3.51%, respectively, and the average daily gain in the Diet 2 group increased (*P* ˂ 0.05) by 14.26% and 11.82%, respectively. Descending trends (*P *= 0.08) were observed in mortality rate in Group 3 and 4 compared with that in Group 1.

**Table 2:** Growth performance of weaned piglets with different diet treatments

Items	Groups				SEM	*P***-**value
	Diet 1	Diet 2	Diet 3	Diet 4		
Initial body weight (kg)	7.03	7.03	7.03	7.02	0.01	0.84
Final body weight (kg)	9.91^b^	10.32^a^	9.97^b^	10.07^ab^	0.06	0.05
Average daily feed intake (g)						
Days 1–7	145.29	157.57	137.86	151.29	3.73	0.29
Days 8–14	303.57	352.71	321.00	325.29	6.72	0.07
Days 1–14	230.57	262.57	236.57	245.00	4.74	0.08
Average daily gain (g)	205.69^b^	235.02^a^	210.18^b^	218.08^ab^	4.13	0.05
Body gain:feed intake (g/g)	0.89	0.90	0.89	0.89	0.01	0.99
Diarrhea rate (%)	1.26	1.85	1.37	1.13	0.18	0.50
Mortality rate (%)	8.79	3.30	2.20	2.20	6.884	0.08

Diet 1: containing 75 mg/kg aureomycin; Diet 2: containing 50 mg/kg aureomycin; Diet 3: containing 50 mg/kg aureomycin and 9 × 10^5^ CFU/g *E. faecium*; Diet 4: containing 50 mg/kg aureomycin and 1.2 × 10^6^ CFU/g *E. faecium*

^a,b^Means with different superscripts in a row differ (*P* < 0.05)

### Diversity of fecal bacterial communities

**Fig. 1:**
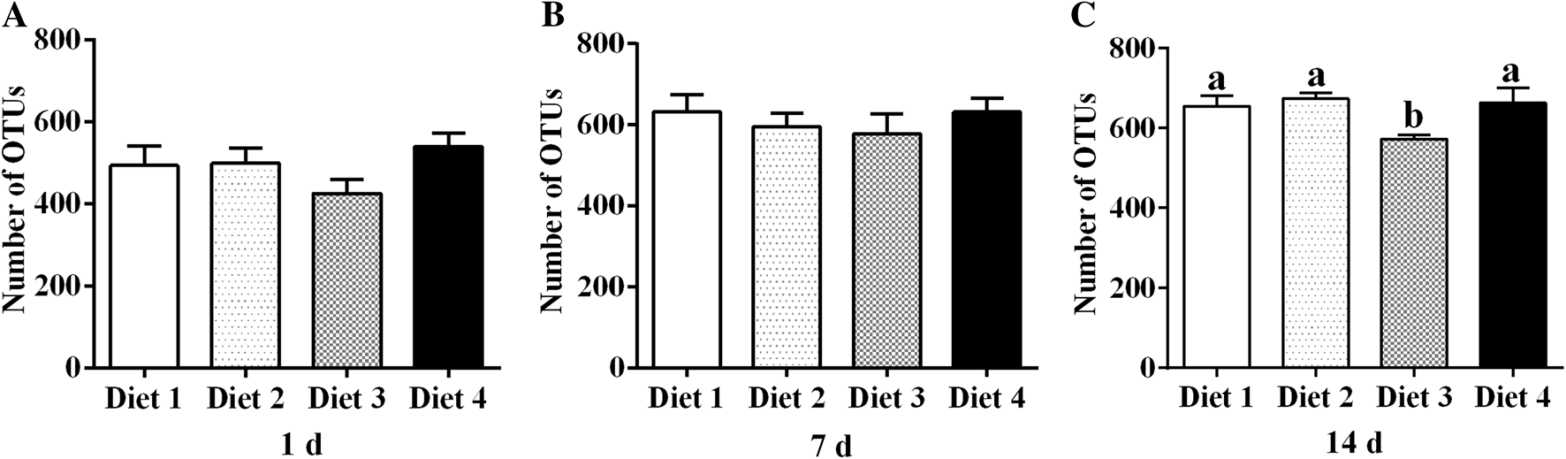
Comparison of the OTUs of the four groups on different experimental days. The number of observed OTUs in fecal samples collected at experimental day 1 (**A**), day 7 (**B**), and day 14 (**C**) sharing the sequence similarity of ≥ 97% is shown. ^a,b^Small letter superscript represents significant difference (*P *< 0.05). Data were statistically analyzed by repeated-measures one-way ANOVA, followed by Duncan’s multiple-range test

Quality control, and chimera removal, 5,769,672 high-quality sequences were obtained from all fecal samples after filtering (Table 3), with an average of 1,442,418 sequences per group and 80,134 per sample. In total, 1852 OTUs were generated. The fecal bacterial community on day 14 in Diet 3 group had fewer OTUs (*P* ˂ 0.05) than those in the other groups (Fig. 1C).

**Table 3:** Raw reads and clean reads among groups

Items	Diet 1	Diet 2	Diet 3	Diet 4
Raw reads (days)				
1	631,816	597,925	506,037	609,058
7	719,889	634,337	596,745	711,415
14	650,988	656,718	571,675	659,874
Clean reads (days)				
1	457,540	456,588	381,826	472,528
7	527,826	503,924	478,440	565,064
14	487,285	489,377	423,904	525,370

1 day, 7 days, and 14 days represent experimental day 1, 7, and 14, respectively. Diet 1: containing 75 mg/kg aureomycin; Diet 2: containing 50 mg/kg aureomycin; Diet 3: containing 50 mg/kg aureomycin and 9 × 10^5^ CFU/g *E. faecium*; Diet 4: containing 50 mg/kg aureomycin and 1.2 × 10^6^ CFU/g *E. faecium*

**Fig. 2:**
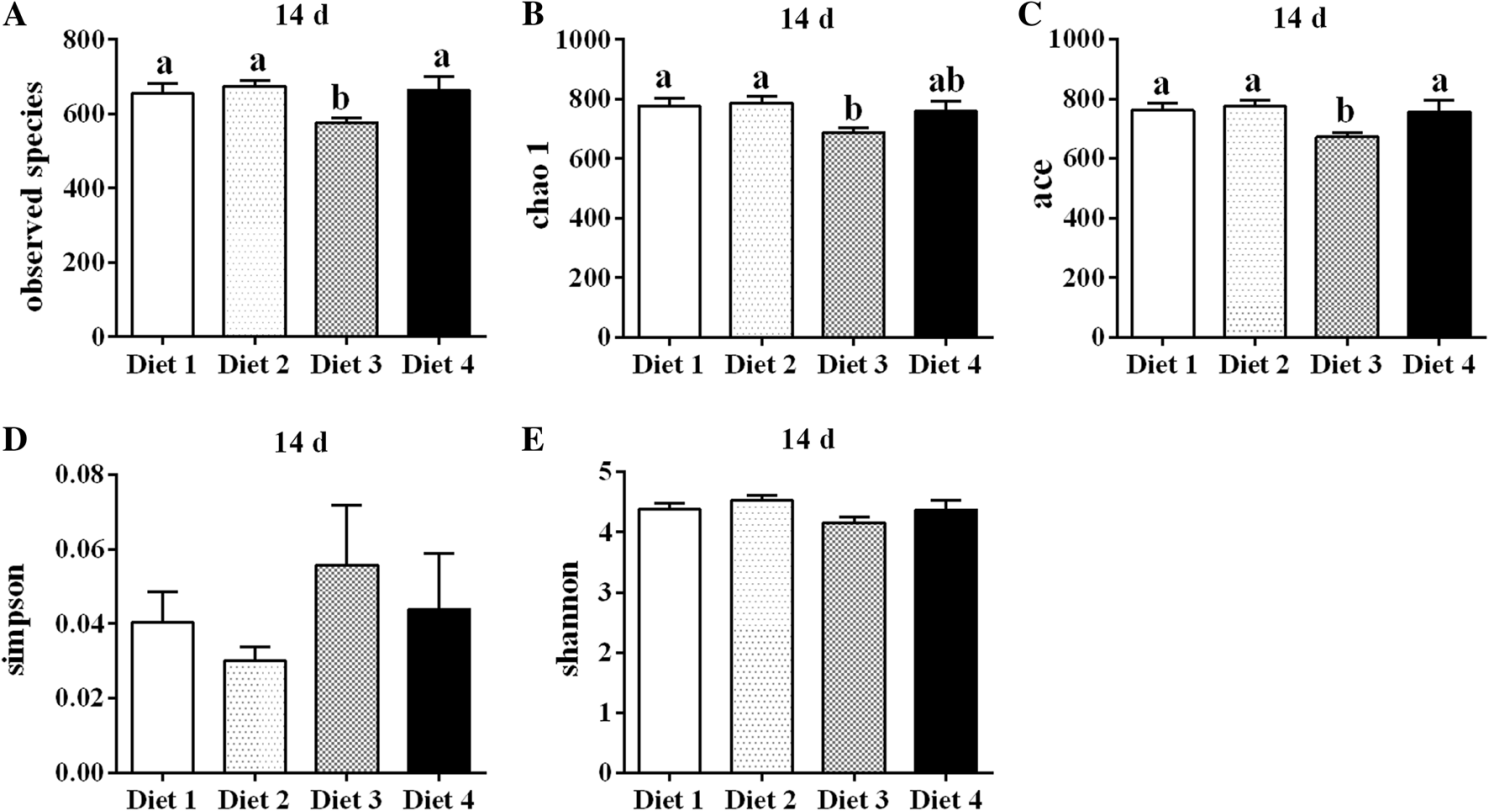
Alpha diversity indices of fecal bacterial communities of weaned piglets on day 14 in different groups.** A**–**E** Observed species, chao 1, ace, simpson, and shannon, respectively. ^a,b^Small letter superscript represents significant difference (*P *< 0.05). Data were statistically analyzed by one-way ANOVA, followed by Duncan’s multiple-range test

**Fig. 3:**
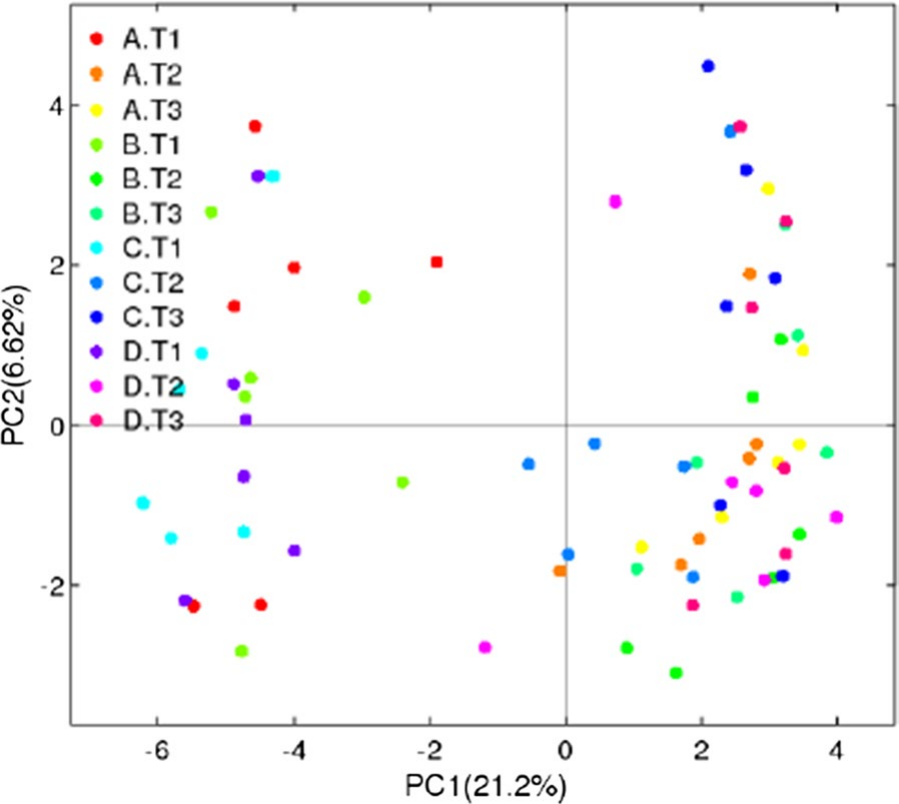
Scatterplot from PCA of OTUs in each fecal sample. A, B, C and D mean fecal samples in diet 1, 2, 3, and 4 group, respectively. T1, T2, and T3 represent fecal samples collected on day 1, day 7, and day 14, respectively

As indicated in Table 4, increases in the Sobs, Chao1, ACE, and Shannon index values and a decrease in the Simpson index value were observed at intervals from day 1 to 14. On day 14, the Sobs, Chao1, ACE, and Shannon index values in Diet 2 group were higher than those in the other groups (Fig. 2A–D). The Diet 3 group exhibited lower (*P *< 0.05) values of the Sobs, Chao1, and ACE indexes than the Diet 1 and Diet 2 groups (Fig. 2A–C). No difference of alpha diversity was found between Diet 1 and Diet 2 groups (*P* > 0.05). The PCA showed that the samples were clustered together on several experimental days (Fig. 3). The rarefaction curve of all samples has reached a stable value (Additional file 1: Figure S1).

**Table 4:** Alpha diversity indices of fecal bacterial communities in weaned piglets at different days

Items^1^	Days			SEM	*P***-**value
	1	7	14		
Sobs					
Diet 1	493.2^b^	632.5^a^	655.8^a^	27.70	0.02
Diet 2	498.7^b^	595.2^a^	674.3^a^	24.04	< 0.01
Diet 3	425.5^b^	578.2^a^	577.7^a^	25.88	0.01
Diet 4	539.6	631.2	664.0	22.54	0.06
Chao1					
Diet 1	613.3	739.8	777.2	30.75	0.07
Diet 2	599.8^b^	686.6^ab^	786.6^a^	27.06	< 0.01
Diet 3	509.2^b^	667.2^a^	687.7^a^	26.86	< 0.01
Diet 4	660.0	743.8	760.4	23.33	0.17
Ace					
Diet 1	594.1^b^	735.0^a^	761.7^a^	30.20	0.04
Diet 2	600.8^b^	692.7^ab^	775.0^a^	26.30	0.02
Diet 3	511.4^b^	669.8^a^	672.5^a^	26.71	< 0.01
Diet 4	650.5	734.7	757.3	23.35	0.14
Shannon					
Diet 1	3.81^b^	4.33^a^	4.37^a^	0.10	0.02
Diet 2	3.89^b^	3.95^b^	4.53^a^	0.11	0.02
Diet 3	3.81	4.04	4.15	0.11	0. 45
Diet 4	4.04	4.21	4.37	0.10	0.42
Simpson					
Diet 1	0.06	0.03	0.04	0.01	0.08
Diet 2	0.06	0.08	0.03	0.02	0.18
Diet 3	0.06	0.07	0.06	0.02	0.89
Diet 4	0.05	0.05	0.04	0.01	0.94

^1^Diet 1: containing 75 mg/kg aureomycin; Diet 2: containing 50 mg/kg aureomycin; Diet 3: containing 50 mg/kg aureomycin and 9 × 10^5^ CFU/g *E. faecium*; Diet 4: containing 50 mg/kg aureomycin and 1.2 × 10^6^ CFU/g *E. faecium*. 1 days, 7 days, and 14 days represent experimental day 1, 7, and 14, respectively

^a,b^Means with different superscripts in a row differ (*P* < 0.05)

### Fecal bacterial community structure

**Fig. 4:**
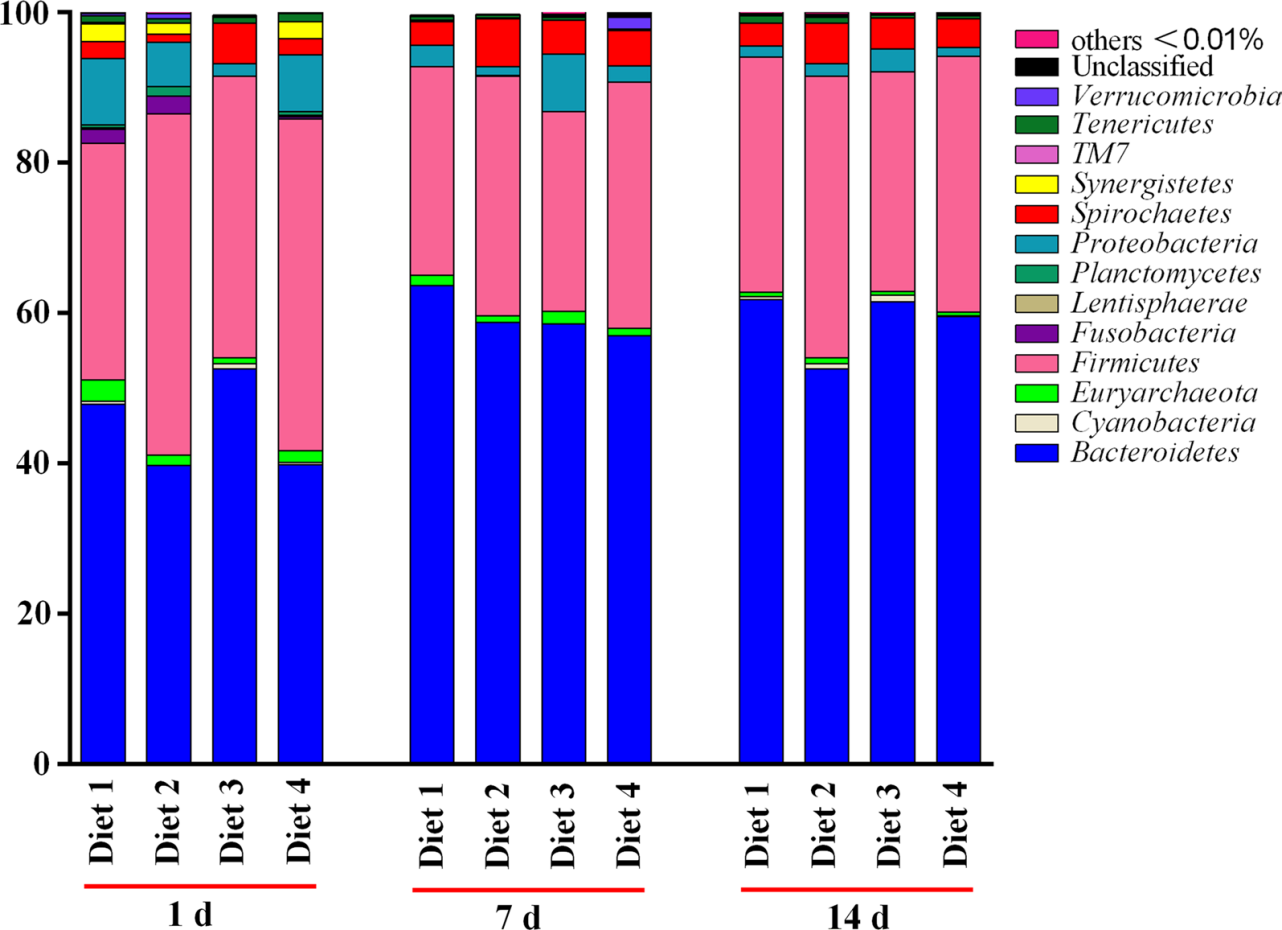
Distribution of bacterial community structure at phylum level in different phases. The relative abundances lower than 0.01% are not shown. 1 day, 7 days, and 14 days represent experimental day 1, 7, and 14, respectively. The relative abundance at phylum level was statistically analyzed through non-parametric Kruskal–Wallis tests

**Fig. 5:**
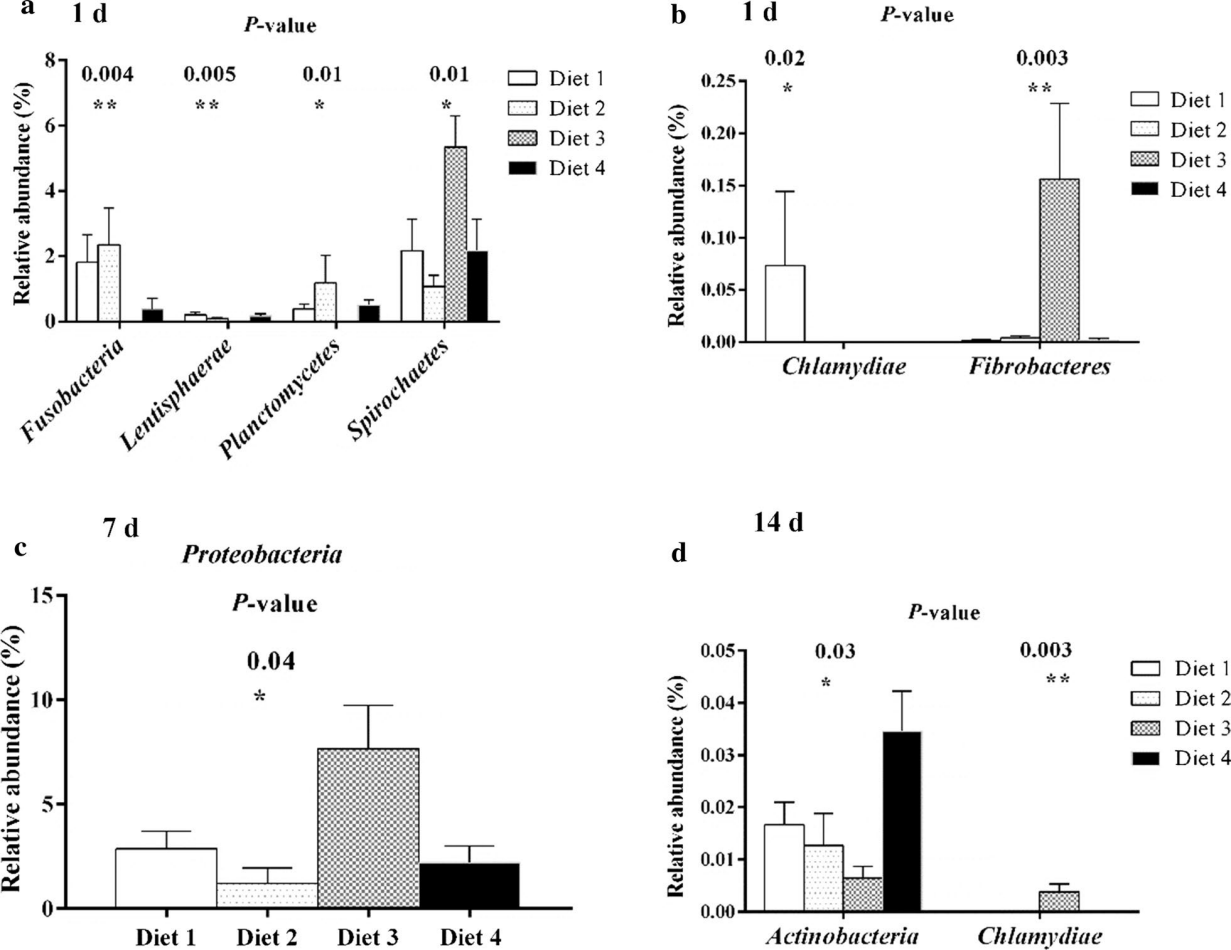
The bacterial abundance of phyla significantly differs in different phases. Only the data whose differences with *P*-values lower than 0.05 are shown. Day 1 (**a**,** b**), day 7 (**c**), and day 14 (**d**) represent experimental day 1, 7, and 14, respectively. *P < 0.05, **P < 0.01

At phylum level, the abundance of seven phyla was ≥ 0.5%: *Bacteroidetes*, *Euryarchaeota*, *Firmicutes*, *Fusobacteria*, *Proteobacteria, Spirochaetes*, and *Synergistetes*. Among them, *Bacteroidetes, Firmicutes, Spirochaetes,* and *Proteobacteria* were the dominant phyla, accounting for more than 95% of the total fecal bacterial community (Fig. 4). The abundance of *Bacteroidetes* was increased whereas that of *Proteobacteria* was decreased from day 1 to 7 and remained stable from day 7 to 14 (Fig. 4). On day 1, the abundance of *Bacteroidetes, Euryarchaeota, Spirochaetes, and Planctomycetes* were higher in the Diet 1 group than in Diet 2 group (Fig. 4), and Diet 3 group exhibited higher (*P* < 0.05) abundances of *Spirochaetes* and *Fibrobacteres* than the other groups (Fig. 5a, b); on days 7 and 14, the abundance of *Proteobacteria* was higher in the Diet 3 group than in the other groups (Fig. 5c), and the abundance of *Firmicutes* was higher in the Diet 2 group than in the Diet 1 group (Fig. 4). Lower abundance of *actinobacteria* was observed (*P* < 0.05) in Diet 3 group when compared to Diet 4 group on day 14 (Fig. 5d).

**Fig. 6:**
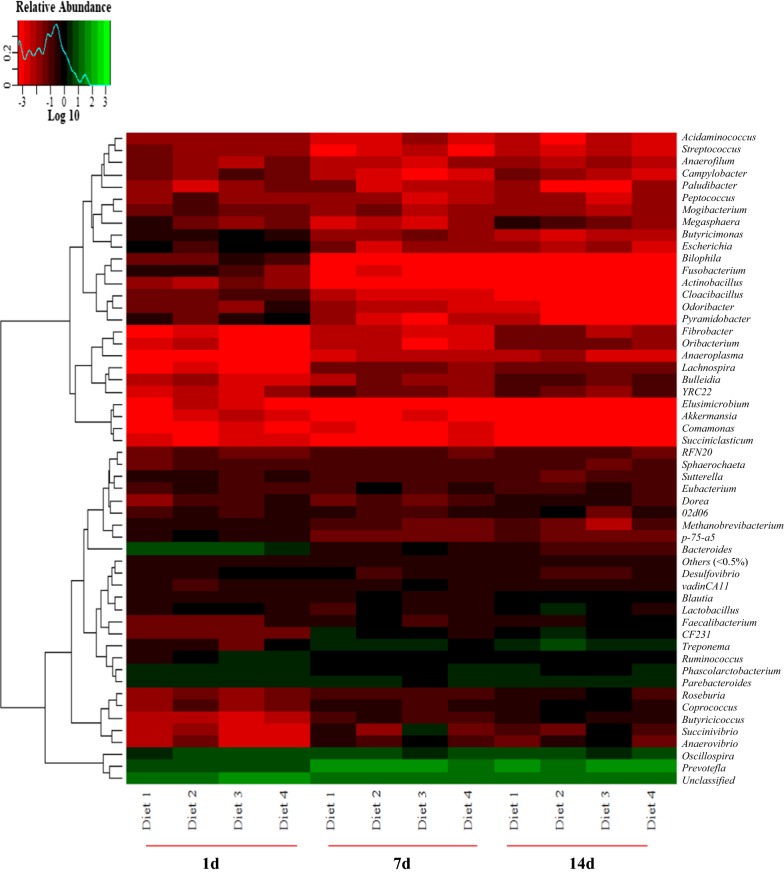
Genus-level taxonomic composition of the bacterial communities of feces in weaned pigs in different phases. The feces with the highest and lowest bacterial levels are shown in green and red respectively. Bacterial genera that rank the top 50 are listed. 1 day, 7 days, and 14 days represent experimental day 1, 7, and 14, respectively

**Fig. 7:**
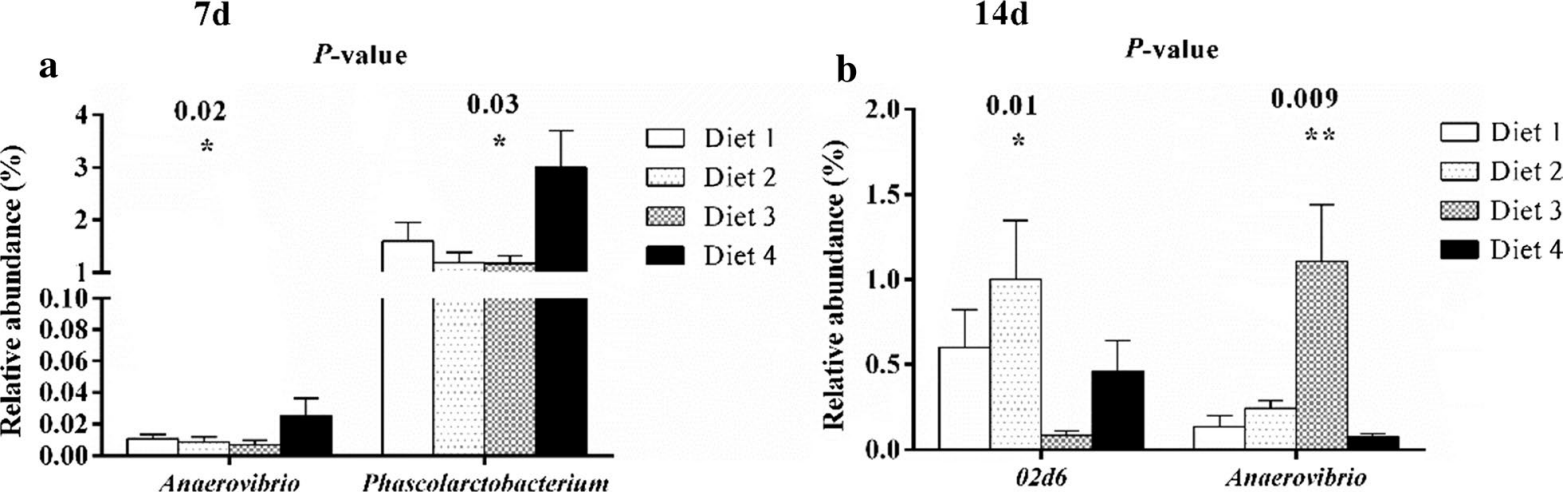
The bacterial abundance of genera significantly differs in different phases. Only the data whose differences with *P*-values are lower than 0.05 are shown. 7 d (**a**) and 14 d (**b**) represent experimental day 7 and 14, respectively. The relative abundance at genus level was statistically analyzed through non-parametric Kruskal–Wallis tests. *P < 0.05, **P < 0.01

Bacterial genera that rank the top 50 are shown in Fig. 6. A higher abundance of *Actinobacillus*, *Bacteroides*, *Butyricimonas*, *Bilophila*, *Escherichia*, *Fusobacterium*, *Odoribacter,* and *Pyramidobacter* and a lower abundance of *Anaeroplasma*, *Anaerovibrio*, *Bulleidia*, *Butyricicoccus*, *Coprococcus*, *Fibrobacter*, *Lachnospira*, *Oribacterium*, *Roseburia*, *Succinivibrio*, and *YRC22* were found on day 1 than those on day 7 and 14. Diet 2 group exhibits the highest abundance of *Lactobacillus* and *Treponema*, and the lowest abundance of *Prevotella* (Fig. 6). On day 7, the highest (*P *< 0.05) abundance of *Anaerovibrio* and *Phascolarctobacterium* was observed in the Diet 4 group (Fig. 7a). On day 14, the abundance of *02d06* was decreased (*P *< 0.05) in the Diet 3 group (Fig. 7b), whereas that of *Anaerovibrio* (Fig. 7b) was increased (*P *< 0.05).

## Discussion

Antibiotics used as growth promoters in feed may bring negative side effects (Chee-Sanford et al. 2001). Therefore, the development of alternatives to antibiotics and a reduction in the use of antibiotics in animal feed are urgently needed. The probiotic bacteria *E. faecium* provides various benefits of health to piglets (Bednorz et al. 2013; Klingspor et al. 2013; Siepert et al. 2014). In the present study, weaned piglets were selected as models to evaluate the effects of a diet with reduced levels of antibiotics and *E. faecium* supplementation for a 14-day intervention period. Significant increases in both final body weight and average daily gain were observed in the Diet 2 group, which indicates that growth performance in weaned piglets fed with a diet with reduced levels of antibiotics was promoted. This is a novel finding, demonstrating that diets with decreasing aureomycin levels ranging from 75 to 50 mg/kg alone had a positive effect on the growth of weaned piglet. However, previous studies showed that dietary supplementation with antibiotics had no effects on final body weight or average daily gain of piglets (Puiman et al. 2013; Yu et al. 2017). In addition, the results of the present study on the effects of dietary antibiotic supplementation are not consistent with those of Wang et al. (2013). Wang et al. claimed that dietary supplementation with 150 mg/kg aureomycin increased the final body weight and weight gain of piglets, and suggested that growth performance in weaned piglets is associated with the dosage of aureomycin used in feed. Interestingly, final body weight and average daily gain of weaned piglets did not change in the Diet 4 group, but were lower in the Diet 3 group than in the Diet 2 group; This does not agree with the findings of Mallo et al. (2010), who proposed that addition of *E. faecium* to the diet promoted the growth and the feed conversion of weaned piglets. Similar results were also found in the treatment of weaned piglets with *E. faecium* (Hu et al. 2015). The discrepancy between these previous studies and the present study could be explained by the differences in the dosages of *E. faecium* used in the diet. Here, the dosages of *E. faecium* in the Diet 3 and 4 groups were 9 × 10^5^ CFU/g and 1.2 × 10^6^ CFU/g, respectively, whereas those of Mallo et al. (2010) and Hu et al. (2015) were 10^6^ CFU/g and 2.5 × 10^6^ CFU/g, respectively. Although no statistically remarkable differences were observed among these groups, the mortality of weaned piglets in the Diet 4 group decreased by 74.9% when compared with those in the Diet 1 group. Overall, these results indicate that a diet with reduced antibiotic levels and *E. faecium* supplementation (Diet 4 group) did not affect the growth of piglets.

The gut microbiota play an important role in the metabolism of hosts (Lippert et al. 2017). In the present study, reduced levels of antibiotics and *E. faecium* supplementation induced changes in the fecal microbiota of weaned piglets. The values of the Sobs, Chao1, ACE, and Shannon indexes in the four diet treatment groups were increased, whereas those of the Simpson index were decreased from day 1 to 14. This proves that the species richness of the community was increased over the course of the experiment. These results agree with those of Frese et al. (2015). In addition, the number of OTUs was significantly reduced in the Diet 3 group on day 14, as were the Sobs, Chao1, and ACE indexes, demonstrating that dietary supplementation with 50 mg/kg aureomycin and 9 × 10^5^ CFU/g *E. faecium* decreased microbial diversity and richness. Microbiota with more diversity have been shown to maintain a more stable ecology and to be favorable for the overall health of animals (Hooper and Macpherson 2010; Hildebrand et al. 2013). Moreover, the microbial richness of heavier piglets is significantly higher than that of lighter piglets (Han et al. 2017), which indicates that microbial richness is associated with the changes in body weight. Therefore, the decreases in the microbial diversity and richness may contribute to explain the lower body weight and average daily gain of weaned piglets in the Diet 3 group. A previous study reported that the alpha diversity was significantly influenced by antibiotic intervention (Tulstrup et al. 2015). However, no changes were found in alpha diversity induced by dietary supplementation with antibiotics in the present study, which is in concord with the results of previous studies (Zhang et al. 2016; Li et al. 2017). This discrepancy could be explained by the differences in the diets and animal models used (Zhao et al. 2015). Tulstrup et al. (2015) used Wistar rats as models which received a daily dosage of 0.5 mL of antibiotic solution containing 60 mg/mL amoxicillin, 8 mg/mL cefotaxime, 8 mg/mL vancomycin and 8 mg/mL metronidazole treatment, whereas piglets are used as models fed with a diet supplemented with 75 mg/kg or 50 mg/kg aureomycin in the present study.

To further illuminate whether changes in the composition of the microbiota were associated with dietary treatment, the distributions of the bacterial community structure at phylum and genus levels were investigated. *Bacteroidetes*, *Firmicutes*, *Spirochaetes*, and *Proteobacteria* were the dominant phyla, which is identical to the conclusions of previous studies (Kong et al. 2016; Yan et al. 2016; Zhang et al. 2016; Mu et al. 2017). In addition, the abundance of *Bacteroidetes* and *Spirochaetes* was increased whereas that of *Proteobacteria* was decreased from day 1 to 7, and the abundance of these phyla kept stable from day 7 to 14. A previous study demonstrated that the abundance of *Bacteroidetes* is associated with protein digestibility (Blackburn and Hobson 1962), and the abundance of *Spirochaetes* is positively correlated with apparent hemicellulose digestibility in piglets (Niu et al. 2015). These results revealed that *Bacteroidetes* and *Spirochaetes* might be involved in the digestion of protein and carbohydrate. Another important finding is that the Diet 3 group exhibited the lowest abundance of *Fusobacteria*, *Lentisphaerae*, and *Planctomycetes* on day 1 and the lowest abundance of *Actinobacteria* on day 14. These findings are similar to the statement that the abundance of *Fusobacteria* and *Lentisphaerae* in piglets feces decreased from age 28 to 150 days (Niu et al. 2015). *Actinobacteria* are considered to be extremely important to the health of animals because of their important roles in the production of antibiotics, antivirals, and enzymes (Newman and Cragg 2007; Tan and Liu 2017). Notably, these results indicate that in Diet 3 Group, the induced body weight loss of weaned piglets was associated with the decreased abundance of bacteria that have a positive effect on the health of hosts. Li et al. (2017) signified that dietary supplementation with 75 mg/kg aureomycin decreased the abundance of *Proteobacteria*, whereas no difference in the abundance of *Proteobacteria* was observed between the Diet 2 and Diet 1 groups in this study.

At genus level, it’s found that taxa belonging to *Firmicutes,* including *Anaerovibrio*, *Coprococcus*, *Oscillospira*, *Phascolarctobacterium*, and *02d06* exhibited marked differences in abundance among the four groups. *Firmicutes* play an important role in starch and fiber degradation (Kim et al. 2011), and increased abundance of *Firmicutes* is associated with obesity and the energy intake of hosts from food in humans (Turnbaugh et al. 2006; Schwiertz et al. 2010). In addition, it is shown that elevated human body weight is associated with a gut microbiota composition characterized by elevated levels of *Firmicutes* (Riva et al. 2017). Here, the abundance of *Firmicutes* was higher in the Diet 2 group than in the Diet 1 group on day 14. Conclusively, the results and findings of the present study suggest that the decrease in growth performance induced by treatment with antibiotics and 9 × 10^5^ CFU/g *E. faecium* was associated with changes in the gut microbiota.

In conclusion, the results of the present study indicate that growth performance in weaned piglets in Diet 4 group was not different from Diet 1 group and 2, whereas growth was reduced in Diet 3 group when compared with Diet 2 group. Taken together, it is concluded that dietary supplementation with 1.2 × 10^6^ CFU/g *E. faecium* instead of partly aureomycin does not affect growth performance, but alters gut microbiota diversity of weaned piglets.

## Additional file

**Additional file 1: Figure S1.** Sample-based rarefaction analysis.

## Abbreviations

OTUs: operational taxonomic units
RDP: Ribosomal Database Project

## References

[CR1] Abhisingha M, Dumnil J, Pitaksutheepong C (2017). Selection of potential probiotic *Lactobacillus* with inhibitory activity against *Salmonella* and fecal coliform bacteria. Probiotics Antimicrob Proteins.

[CR2] Andreas P, Farfan-Lopez C, Mora F, Rondon Y, Rossini M, Araque H (2016). Effect of using mannoproteins and antibiotics as growth promoters in diets for weaned piglets on performance. Revista Cientifica-Facultad De Ciencias Veterinarias.

[CR3] Bednorz C, Guenther S, Oelgeschlager K, Kinnemann B, Pieper R, Hartmann S, Tedin K, Semmler T, Neumann K, Schierack P, Bethe A, Wieler LH (2013). Feeding the probiotic *Enterococcus faecium* strain NCIMB 10415 to piglets specifically reduces the number of *Escherichia coli* pathotypes that adhere to the gut mucosa. Appl Environ Microbiol.

[CR4] Blackburn TH, Hobson PN (1962). Further studies on the isolation of proteolytic bacteria from the sheep rumen. J Gen Microbiol.

[CR5] Busing K, Zeyner A (2015). Effects of oral *Enterococcus faecium* strain DSM 10663 NCIMB 10415 on diarrhoea patterns and performance of sucking piglets. Benef Microbes.

[CR6] Caporaso JG, Kuczynski J, Stombaugh J, Bittinger K, Bushman FD, Costello EK, Fierer N, Pena AG, Goodrich JK, Gordon JI, Huttley GA, Kelley ST, Knights D, Koenig JE, Ley RE, Lozupone CA, McDonald D, Muegge BD, Pirrung M, Reeder J, Sevinsky JR, Turnbaugh PJ, Walters WA, Widmann J, Yatsunenko T, Zaneveld J, Knight R (2010). QIIME allows analysis of high-throughput community sequencing data. Nat Methods.

[CR7] Casewell M, Friis C, Marco E, McMullin P, Phillips I (2003). The European ban on growth-promoting antibiotics and emerging consequences for human and animal health. J Antimicrob Chemother.

[CR8] Chee-Sanford JC, Aminov RI, Krapac IJ, Garrigues-Jeanjean N, Mackie RI (2001). Occurrence and diversity of tetracycline resistance genes in lagoons and groundwater underlying two swine production facilities. Appl Environ Microbiol.

[CR9] DeSantis TZ, Hugenholtz P, Larsen N, Rojas M, Brodie EL, Keller K, Huber T, Dalevi D, Hu P, Andersen GL (2006). Greengenes, a chimera-checked 16S rRNA gene database and workbench compatible with ARB. Appl Environ Microbiol.

[CR10] Edgar RC (2013). UPARSE: highly accurate OTU sequences from microbial amplicon reads. Nat Methods.

[CR11] Frese SA, Parker K, Calvert CC, Mills DA (2015). Diet shapes the gut microbiome of pigs during nursing and weaning. Microbiome.

[CR12] Gresse R, Chaucheyras-Durand F, Fleury MA, Van de Wiele T, Forano E, Blanquet-Diot S (2017). Gut microbiota dysbiosis in postweaning piglets: understanding the keys to health. Trends Microbiol.

[CR13] Han GG, Lee JY, Jin GD, Park J, Choi YH, Chae BJ, Kim EB, Choi YJ (2017). Evaluating the association between body weight and the intestinal microbiota of weaned piglets via 16S rRNA sequencing. Appl Microbiol Biotechnol.

[CR14] Hildebrand F, Nguyen TL, Brinkman B, Yunta RG, Cauwe B, Vandenabeele P, Liston A, Raes J (2013). Inflammation-associated enterotypes, host genotype, cage and inter-individual effects drive gut microbiota variation in common laboratory mice. Genome Biol.

[CR15] Hooper LV, Macpherson AJ (2010). Immune adaptations that maintain homeostasis with the intestinal microbiota. Nat Rev Immunol.

[CR16] Hu Y, Dun Y, Li S, Zhang D, Peng N, Zhao S, Liang Y (2015). Dietary *Enterococcus faecalis* LAB31 improves growth performance, reduces diarrhea, and increases fecal *Lactobacillus* number of weaned piglets. PLoS ONE.

[CR17] Hu QL, Li SS, Zhang YW, Zhuo Z, Feng J (2017). Phytosterols on growth performance, antioxidant enzymes and intestinal morphology in weaned piglets. J Sci Food Agric.

[CR18] Kahrstrom CT, Pariente N, Weiss U (2016). Intestinal microbiota in health and disease. Nature.

[CR19] Kim M, Morrison M, Yu Z (2011). Status of the phylogenetic diversity census of ruminal microbiomes. FEMS Microbiol Ecol.

[CR20] Klingspor S, Martens H, Caushi D, Twardziok S, Aschenbach JR, Lodemann U (2013). Characterization of the effects of *Enterococcus faecium* on intestinal epithelial transport properties in piglets. J Anim Sci.

[CR21] Kong XF, Ji YJ, Li HW, Zhu Q, Blachier F, Geng MM, Chen W, Yin YL (2016). Colonic luminal microbiota and bacterial metabolite composition in pregnant Huanjiang mini-pigs: effects of food composition at different times of pregnancy. Sci Rep.

[CR22] Lan RX, Kim IH (2017). Effects of dietary supplementation with a probiotic (*Enterococcus faecium* DSM 7134) on growth performance, nutrient digestibility, and gut health status in weaning pigs. J Sci Food Agric.

[CR23] Li M, Monaco MH, Wang M, Comstock SS, Kuhlenschmidt TB, Fahey GC, Miller MJ, Kuhlenschmidt MS, Donovan SM (2014). Human milk oligosaccharides shorten rotavirus-induced diarrhea and modulate piglet mucosal immunity and colonic microbiota. ISME J.

[CR24] Li H, Liang T, Chu Q, Xu F, Li Y, Fu L, Zhou B (2017). Effects of several in-feed antibiotic combinations on the abundance and diversity of fecal microbes in weaned pigs. Can J Microbiol.

[CR25] Lippert K, Kedenko L, Antonielli L, Kedenko I, Gemeier C, Leitner M, Kautzky-Willer A, Paulweber B, Hackl E (2017). Gut microbiota dysbiosis associated with glucose metabolism disorders and the metabolic syndrome in older adults. Benef Microbes.

[CR26] Liu H, Zhang J, Zhang S, Yang F, Thacker PA, Zhang G, Qiao S, Ma X (2014). Oral administration of *Lactobacillus fermentum* I5007 favors intestinal development and alters the intestinal microbiota in formula-fed piglets. J Agric Food Chem.

[CR27] Magoc T, Salzberg SL (2011). FLASH: fast length adjustment of short reads to improve genome assemblies. Bioinformatics.

[CR28] Mallo JJ, Rioperez J, Honrubia P (2010). The addition of *Enterococcus faecium* to diet improves piglet’s intestinal microbiota and performance. Livest Sci.

[CR29] Mu C, Yang Y, Su Y, Zoetendal EG, Zhu W (2017). Differences in microbiota membership along the gastrointestinal tract of piglets and their differential alterations following an early-life antibiotic intervention. Front Microbiol.

[CR30] Newman DJ, Cragg GM (2007). Natural products as sources of new drugs over the last 25 years. J Nat Prod.

[CR31] Niu Q, Li P, Hao S, Zhang Y, Kim SW, Li H, Ma X, Gao S, He L, Wu W, Huang X, Hua J, Zhou B, Huang R (2015). Dynamic distribution of the gut microbiota and the relationship with apparent crude fiber digestibility and growth stages in pigs. Sci Rep.

[CR32] Puiman P, Stoll B, Molbak L, de Bruijn A, Schierbeek H, Boye M, Boehm G, Renes I, van Goudoever J, Burrin D (2013). Modulation of the gut microbiota with antibiotic treatment suppresses whole body urea production in neonatal pigs. Am J Physiol Gastrointest Liver Physiol.

[CR33] Riva A, Borgo F, Lassandro C, Verduci E, Morace G, Borghi E, Berry D (2017). Pediatric obesity is associated with an altered gut microbiota and discordant shifts in *Firmicutes* populations. Environ Microbiol.

[CR34] Schwiertz A, Taras D, Schafer K, Beijer S, Bos NA, Donus C, Hardt PD (2010). Microbiota and SCFA in lean and overweight healthy subjects. Obesity.

[CR35] Sen T, Cawthon CR, Ihde BT, Hajnal A, DiLorenzo PM, de La Serre CB, Czaja K (2017). Diet-driven microbiota dysbiosis is associated with vagal remodeling and obesity. Physiol Behav.

[CR36] Siepert B, Reinhardt N, Kreuzer S, Bondzio A, Twardziok S, Brockmann G, Nockler K, Szabo I, Janczyk P, Pieper R, Tedin K (2014). *Enterococcus faecium* NCIMB 10415 supplementation affects intestinal immune-associated gene expression in post-weaning piglets. Vet Immunol Immunopathol.

[CR37] Sonnenburg JL, Backhed F (2016). Diet-microbiota interactions as moderators of human metabolism. Nature.

[CR38] Tan GY, Liu T (2017). Rational synthetic pathway refactoring of natural products biosynthesis in actinobacteria. Metab Eng.

[CR39] Taras D, Vahjen W, Macha M, Simon O (2006). Performance, diarrhea incidence, and occurrence of *Escherichia coli* virulence genes during long-term administration of a probiotic *Enterococcus faecium* strain to sows and piglets. J Anim Sci.

[CR40] Tulstrup MV, Christensen EG, Carvalho V, Linninge C, Ahrne S, Hojberg O, Licht TR, Bahl MI (2015). Antibiotic treatment affects intestinal permeability and gut microbial composition in Wistar rats dependent on antibiotic class. PLoS ONE.

[CR41] Turnbaugh PJ, Ley RE, Mahowald MA, Magrini V, Mardis ER, Gordon JI (2006). An obesity-associated gut microbiome with increased capacity for energy harvest. Nature.

[CR42] van den Bogaard AE, Stobberingh EE (2000). Epidemiology of resistance to antibiotics: links between animals and humans. Int J Antimicrob Agents.

[CR43] Vondruskova H, Slamova R, Trckova M, Zraly Z, Pavlik I (2010). Alternatives to antibiotic growth promoters in prevention of diarrhoea in weaned piglets: a review. Vet Med.

[CR44] Wang AN, Cai CJ, Zeng XF, Zhang FR, Zhang GL, Thacker PA, Wang JJ, Qiao SY (2013). Dietary supplementation with *Lactobacillus fermentum* I5007 improves the anti-oxidative activity of weanling piglets challenged with diquat. J Appl Microbiol.

[CR45] Wang YB, Du W, Fu AK, Zhang XP, Huang Y, Lee KH, Yu K, Li WF, Li YL (2016). Intestinal microbiota and oral administration of *Enterococcus faecium* associated with the growth performance of new-born piglets. Benef Microbes.

[CR46] Wei HK, Xue HX, Zhou ZX, Peng J (2017). A carvacrol–thymol blend decreased intestinal oxidative stress and influenced selected microbes without changing the messenger RNA levels of tight junction proteins in jejunal mucosa of weaning piglets. Animal.

[CR47] Yan H, Diao H, Xiao Y, Li W, Yu B, He J, Yu J, Zheng P, Mao X, Luo Y, Zeng B, Wei H, Chen D (2016). Gut microbiota can transfer fiber characteristics and lipid metabolic profiles of skeletal muscle from pigs to germ-free mice. Sci Rep.

[CR48] Yu M, Zhang C, Yang Y, Mu C, Su Y, Yu K, Zhu W (2017). Long-term effects of early antibiotic intervention on blood parameters, apparent nutrient digestibility, and fecal microbial fermentation profile in pigs with different dietary protein levels. J Anim Sci Biotechnol.

[CR49] Zeng B, Zhao J, Guo W, Zhang S, Hua Y, Tang J, Kong F, Yang X, Fu L, Liao K, Yu X, Chen G, Jin L, Shuai S, Yang J, Si X, Ning R, Mishra S, Li Y (2017). High-altitude living shapes the skin microbiome in humans and pigs. Front Microbiol.

[CR50] Zeyner A, Boldt E (2006). Effects of a probiotic *Enterococcus faecium* strain supplemented from birth to weaning on diarrhoea patterns and performance of piglets. J Anim Physiol Anim Nutr.

[CR51] Zhang D, Ji H, Liu H, Wang S, Wang J, Wang Y (2016). Changes in the diversity and composition of gut microbiota of weaned piglets after oral administration of *Lactobacillus* or an antibiotic. Appl Microbiol Biotechnol.

[CR52] Zhao W, Wang Y, Liu S, Huang J, Zhai Z, He C, Ding J, Wang J, Wang H, Fan W, Zhao J, Meng H (2015). The dynamic distribution of porcine microbiota across different ages and gastrointestinal tract segments. PLoS ONE.

