# Supplementary figures and images for: Effects of dietary supplementation of probiotic Enterococcus faecium on growth performance and gut microbiota in weaned piglets
Source: AMB Express. 2019 Mar 1;9:33. doi: 10.1186/s13568-019-0755-z (PMC6397275; doi:10.1186/s13568-019-0755-z)

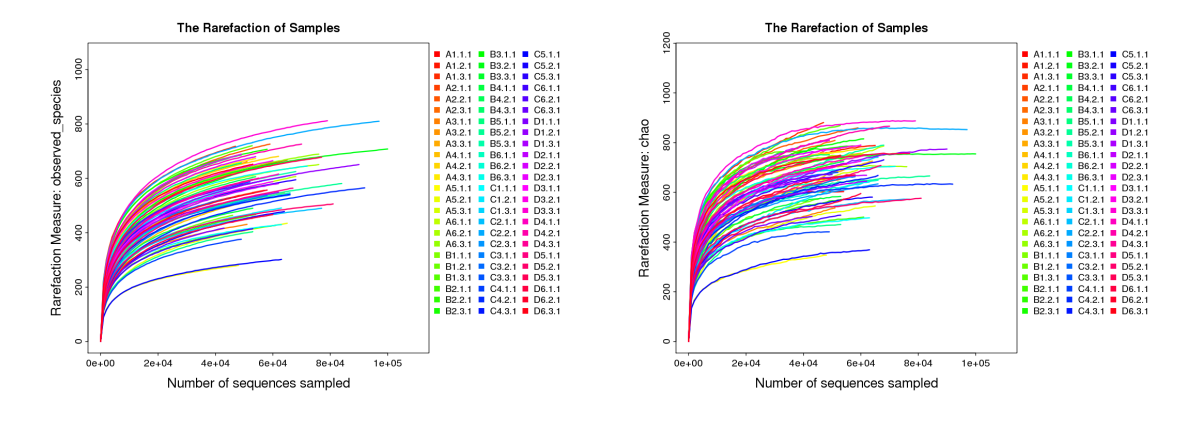

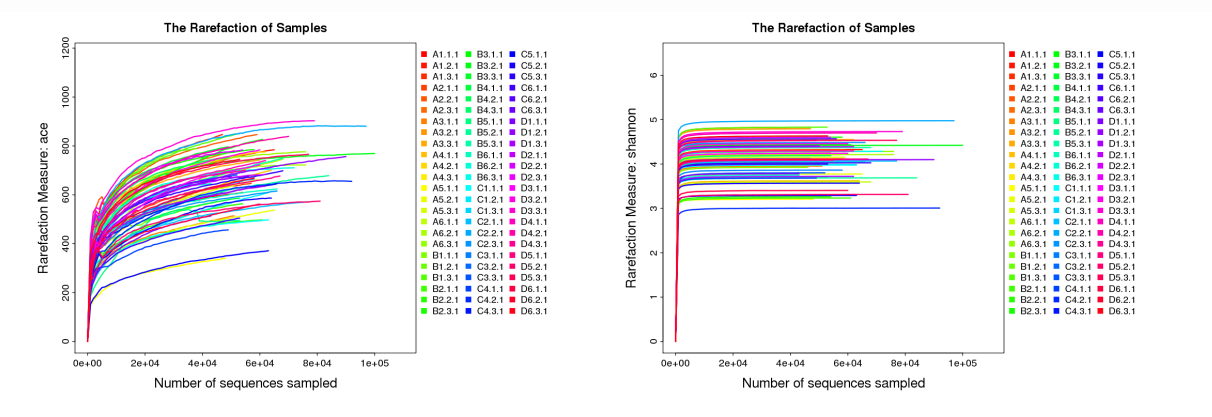

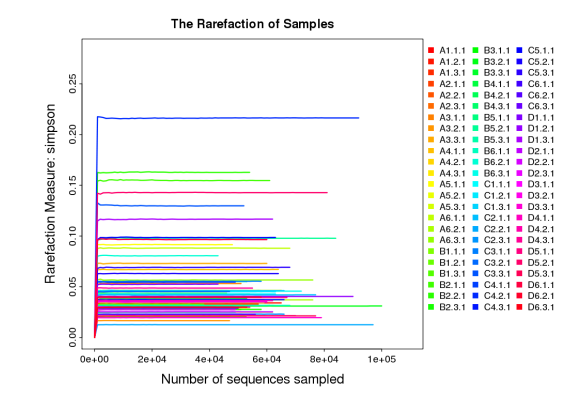


Fig S1**.** Sample-based rarefaction analysis.

Supplement: Supplementary file 1 — Additional file 1: Figure S1. Sample-based rarefaction analysis. [file 13568_2019_755_MOESM1_ESM.docx]
